# Cytotoxicity of quantum dots and graphene oxide to erythroid cells and macrophages

**DOI:** 10.1186/1556-276X-8-198

**Published:** 2013-04-30

**Authors:** Guangbo Qu, Xiaoyan Wang, Zhe Wang, Sijin Liu, Guibing Jiang

**Affiliations:** 1 State Key Laboratory of Environmental Chemistry and Ecotoxicology, Research Center for Eco-Environmental Sciences, Chinese Academy of Sciences, Beijing 100085, China

**Keywords:** Quantum dots, Graphene oxidative, Erythroid cells, Macrophages, ROS, Apoptosis

## Abstract

Great concerns have been raised about the exposure and possible adverse influence of nanomaterials due to their wide applications in a variety of fields, such as biomedicine and daily lives. The blood circulation system and blood cells form an important barrier against invaders, including nanomaterials. However, studies of the biological effects of nanomaterials on blood cells have been limited and without clear conclusions thus far. In the current study, the biological influence of quantum dots (QDs) with various surface coating on erythroid cells and graphene oxide (GO) on macrophages was closely investigated. We found that QDs posed great damage to macrophages through intracellular accumulation of QDs coupled with reactive oxygen species generation, particularly for QDs coated with PEG-NH_2_. QD modified with polyethylene glycol-conjugated amine particles exerted robust inhibition on cell proliferation of J744A.1 macrophages, irrespective of apoptosis. Additionally, to the best of our knowledge, our study is the first to have demonstrated that GO could provoke apoptosis of erythroid cells through oxidative stress in E14.5 fetal liver erythroid cells and *in vivo* administration of GO-diminished erythroid population in spleen, associated with disordered erythropoiesis in mice.

## Background

Of the popular nanomaterials, quantum dots (QDs) and graphene have promising applications in various fields; however, the cytotoxicty of these nanomaterials is also largely concerned [1,2]. To date, a few studies have revealed that QDs and graphene posed harm to a spectrum of organisms and cells [3-6]. Blood cells are a large group of cells that play critical roles in many physiological and pathological processes. Of the blood cells, erythrocytes are responsible for carrying oxygen, carbon dioxide, and other wastes; whereas, macrophages are part of the immune system responsible for inflammation and the clearance of pathogens [7]. Erythropoiesis is a highly dynamic process that produces numerous new red blood cells (RBCs), which requires a large amount of iron [8,9]. Senescent erythrocytes undergo phagocytosis by macrophages, and iron is released into the circulation for erythropoiesis upon erythropoietic demand [10]. Thus, erythrocytes and macrophages are essentially involved in governing the balance of erythropoiesis and iron recycling in the body. Thus far, limited work has been performed in blood cells in evaluating the biosafety of QDs and graphene.

Previous studies have documented that QDs could transport through the plasma membrane of RBCs, exerting potential impairment on the survival or function of RBCs [11]. Our own studies have demonstrated that QDs engulfed by macrophages in spleen could cause impairment to macrophages, which triggered the accumulation of aged RBCs in spleen with splenomegaly [12]. A few other studies have also suggested that graphene or graphene oxide (GO) might impose toxicity to RBCs through hemolysis and incur cell death and cytoskeleton destruction to macrophages [13-16]. To date, the cytotoxicity and related mechanisms of QDs and graphene still remain inconclusive for blood cells due to limited data. To this end, in the current study, we embarked on the cytotoxicity of QDs with different surface modifications to macrophages and GO to erythroid cells. Overall, we demonstrated significant adverse effects of QDs on macrophages and GO on erythrocytes.

## Methods

### Nanomaterials

QDs with the same core Cd/Te coated with Sn/S and the same diameter (approximately 4 nm) modified with polyethylene glycol (PEG) (QD-PEG), PEG-conjugated amine (QD-PEG-NH_2_), or PEG-conjugated carboxyl groups (QD-PEG-COOH) were purchased from Wuhan Jiayuan Quantum Dots Co., Ltd. (Wuhan, China) [12,17]. The evaluation of the fluorescence spectrum indicated that the maximum emission wavelength for QDs used here was around 605 nm, indicative of red light. GO was synthesized using the Hummers method with minor revisions as previously described [18]. The size of GO was 300 to 1,000 nm, and the thickness was approximately 1 nm [18]. GO suspension was stable for at least 1 month. GO suspension was diluted in phosphate buffered saline (PBS) for the following experiments.

### Animal experiments

Regarding the GO administration *in vivo*, 6-week-old BALB/C male mice were intraperitoneally injected with 200 μl GO suspension at a concentration of 1 mg/ml (10 mg/kg body weight) every 3 days for 3 weeks. Control mice received PBS only. Twenty four h after the final administration, blood was collected via the heart, and complete blood count (CBC) analysis was carried out using a whole blood analyzer at Peking University Health Center. After the mice were sacrificed, organs were collected.

### Characterization of cell population in organs by fluorescence-activated cell sorting

After perfusion with saline, livers were perfused with 0.05% collagenase and then minced and resuspended in 0.05 g/ml collagenase type IV (Sigma-Aldrich, St. Louis, MO, USA) in Hank's balanced salt buffer [18]. The samples were then incubated in the solution without either cadmium or magnesium for enzymatic digestion at 37°C for 30 min. The digested samples were passed through 70 μm filters. The cells were resuspended in PBS and then incubated with fluorescein isothiocyanate (FITC)-conjugated anti-F4/80 mAb (eBioscience Inc., San Diego, CA, USA) for the selection of macrophage population. Phycoerythrin (PE)-conjugated anti-Ter119 mAb (BD Pharmingen, Franklin Lakes, NJ, USA) was applied to cell suspension for erythroid cell selection. After washing, the cells were analyzed on a fluorescence-activated cell sorting (FACS) Calibur™ (BD Biosciences, San Jose, CA, USA). Splenocytes were similarly prepared from the spleen for FACS analysis.

### Cell culture and treatment

Mouse J774A.1 (purchased from the Shanghai Cell Bank of Type Culture Collection of the Chinese Academy of Sciences, Shanghai, China) were cultured in DMEM (Hyclone, Thermo Fisher Scientific, Waltham, MA, USA), supplemented with 10% fetal bovine serum (Gibco, Carlsbad, CA, USA) and 100 U/ml penicillin/streptomycin (Gibco). E14.5 fetal liver cells were isolated and cultured as described [19].

### Determination of cadmium mass

Regarding the assessment of intracellular cadmium mass, J774A.1 cells cultured in 10-cm plates were exposed to QDs for 24 h. Thereafter, the cells were collected and washed with PBS for three times, and cells were digested with HNO_3_ and H_2_O_2_ (3:2, *v*/*v*) by microwave-assisted extraction. After the removal of acid, the digested samples were diluted to 5 ml, and Cd mass was assessed using inductively coupled plasma mass spectrometry (ICP-MS) (Agilent 7500, Santa Clara, CA, USA) according to the protocol as previously described [20]. A series of cadmium standard solutions (10, 5, 2, 1, 0.5, 0.2, and 0 ng/g) were prepared to conduct a standard curve for the calibration of Cd concentration.

### Cell proliferation assay

Cell proliferation was evaluated by the BrdU incorporation assay (Roche, Penzberg, Germany). Briefly, the cells were seeded in 96-well plates with 5.0 × 10^4^ cells per well in 100 μl. The cells were starved in 1% FBS serum medium overnight. The cells were then treated with 47 μg/ml QDs for 48 h, and cell growth was examined according to the instructions provided by the manufacturer.

### Confocal laser scanning microscopy

After exposure to 47 μg/ml QDs for 24 h, the cells were fixed by formaldehyde, followed by a wash with 1% Triton X-100 in PBS. FITC-conjugated phalloidin (Molecular Probes, Invitrogen Corporation, Grand Island, NY, USA) was used to stain filamentous actin (F-actin), and nuclei were counterstained with 4',6-diamidino-2-phenylindole (DAPI) (blue) (Molecular Probes). Laser scanning confocal microscopy was performed to image cells as previously described [21].

### Reactive oxygen species measurement

After preincubation with 10 μM 2'-7'-Dichlorodihydrofluorescein diacetate (DCFH-DA) (Sigma-Aldrich) for 30 min, the J774A.1 cells seeded in 24 well-plate (1.0 × 10^5^ per well) were treated with QDs at 47 μg/ml for 6 h. After treatment, the emission spectra of dichlorodihydrofluorescein (DCF) fluorescence at 525 nM were measured using FACS Calibur™ (BD Biosciences). The E14.5 fetal cells were similarly cultured and preincubated with DCFH-DA. Thereafter, the cells were washed with PBS, and treated with 10, 20, 40, and 80 μg/ml GO for 15 min, 0.5 h, 1 h, and 6 h, respectively, followed by DCF fluorescence determination.

### Cell death by fluorescence-activated cell sorting analysis

For apoptosis analysis of erythroid cells from spleen, splenic cell suspension was co-stained with PE-conjugated anti-Ter119 Ab, FITC-conjugated Annexin V and 7-amino-actinomycin D (7AAD). The cell death of erythroid cells was determined with the channels of Annexin V fluorescence and 7AAD fluorescence by gating Ter119^+^ cells. With respect to J774A.1 cells, after exposure to QDs for 24 h, the cells were subject to FITC-conjugated Annexin V and propidium iodide (PI) staining. Apoptotic and necrotic cells were assessed by FACS as described previously [22]. The E14.5 fetal liver cells were treated with 20 μg/ml GO for 18 h, and cell death was then similarly examined.

### Statistical analysis

One-way analysis of variance (ANOVA) was employed to assess the mean difference among the groups compared to control. The difference between the two groups was analyzed with two-tailed Student's *t* test. All experimental data were shown in mean ± SD. *P* < 0.05 was considered to be statistically significant.

All animal care and surgical procedures were approved by the Animal Ethics Committee at the Research Center for Eco-Environmental Sciences, Chinese Academy of Sciences.

## Results and discussion

**Figure 1:**
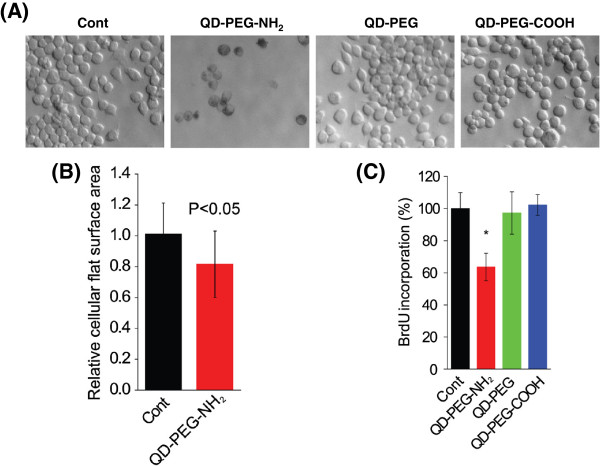
**Biological influence of QDs on J774A.1 cells. (A)** Bright field images of J774A.1 cells treated with QDs with different surface modifications at 47 μg/ml for 5 days (×40). **(B)** The bar graph represents the relative cellular flat surface area of J774A.1 cells treated with 47 μg/ml QDs coated with PEG-NH_2_ for 5 days (*n* = 50). **(C)** Cell proliferation was evaluated with the BrdU incorporation assay upon treatment with 47 μg/ml QDs with different surface modifications for 24 h (*n* = 6). Asterisk indicates *P* < 0.001.

**Figure 2:**
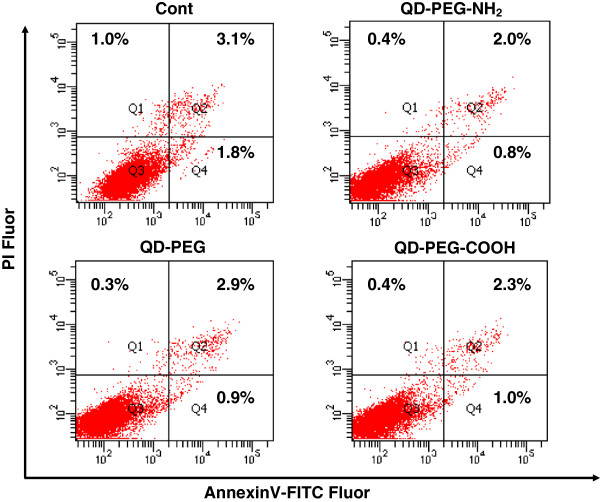
**Cell death of J774A.1 cells in response to QD treatment.** Representative images of cell death of J774A.1 cells after 24-h treatment with 47 μg/ml QDs with different surface modifications assessed by FACS analysis with FITC Annexin V and PI staining.

Our recent study demonstrated that QDs coated with PEG could impair the morphology and the ability of J774A.1 macrophages in phagocytosis; however, no significant cytotoxicity in survival was observed in these cells [12]. We then studied the potential effect of surface modification on QD-mediated cytotoxicity to macrophages. A small number of J774A.1 cells in 6-well plates (5.0 × 10^4^/well) were seeded and treated with QD particles precoated with PEG, PEG-NH_2_, or PEG-COOH, and the cells were then observed for 5 days. As shown in Figure 1A, the number of cells upon QD-PEG or QD-PEG-COOH treatment was 21.4 × 10^4^ and 19.3 × 10^4^, similar to that in the control (*P* > 0.05); however, the number of cells treated with QD-PEG-NH_2_ was 4.7 × 10^4^, much lower than that in the control (*P* < 0.001). Moreover, the relative cellular flat surface area was measured with the Image-Pro-Plus software (Media Cybernetics, Rockville, MD, USA), and the results indicated that the average size per cell was reduced by approximately 20% compared to the control (Figure 1A,B, *P* < 0.05). To tease apart the mechanisms responsible for the cytotoxicity of QD-PEG-NH_2_ to J774A.1 macrophages, we individually assessed cell proliferation and apoptosis. The BrdU incorporation assay indicated that the cell division of J774A.1 cells upon QD-PEG-NH_2_ exposure for 24 h was greatly diminished by approximately 40% compared to the control (*P* < 0.001), and cell growth was rarely affected in cells treated with QD-PEG or QD-PEG-COOH (Figure 1C), suggesting a robust inhibition of QD-PEG-NH_2_ on cell proliferation. To exclude possible involvement of cell death induced by QD-PEG-NH_2_, we therefore surveyed apoptosis and necrosis with FACS analysis after PI and FITC-conjugated Annexin V staining. Annexin V binds to phosphatidylserine that localizes on the outer surface of cell membrane, which is an early event in apoptosis and PI stains nucleus of necrotic cells [23]. As shown in Figure 2, the proportion of cells representing early apoptosis (Q4 region, Annexin V^+^PI^−^), necrosis (Q1 region, Annexin V^-^PI^+^), and late apoptosis or necrosis (Q2 region, Annexin V^+^PI^+^) remained similar among different treatments after 24 h compared to the control, demonstrating that QDs with these kinds of surface modifications exerted no cell death to J774A.1 cells.

**Figure 3:**
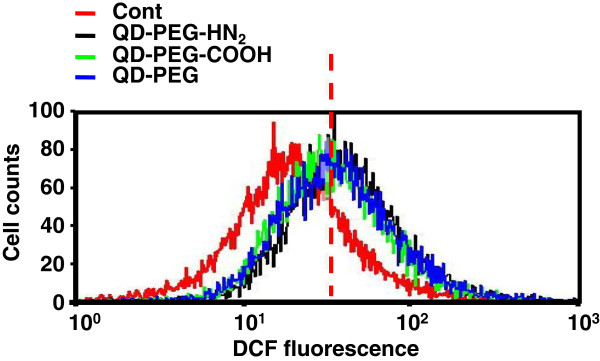
**ROS generation upon QD treatment in J774A.1 cells.** FACS analysis of the relative intensity of DCF fluorescence reflecting intracellular ROS level after exposure to QDs with different surface modifications at 47 μg/ml in fetal liver cells for 6 h.

It has also been reported that QD treatment could cause impairment of cell growth through induction of reactive oxygen species (ROS) [24]. We thus assessed intracellular ROS generation in J774A.1 cells upon QD treatment with FACS analysis of DCF fluorescence. As shown in Figure 3, an increase of intracellular ROS could be determined in cells upon 6-h treatment similarly with QD-PEG, QD-PEG-COOH, and QD-PEG-NH_2_ particles, compared to the control (Figure 3, *P* < 0.05). The increase of ROS generation was close among the three types of QDs (Figure 3, *P* > 0.05). These data together indicated that ROS production was independent of surface modification on QDs, and ROS did not account for the cytotoxicity of QD-PEG-NH_2_ particles in repressing the proliferation of J774A.1 cells.

**Figure 4:**
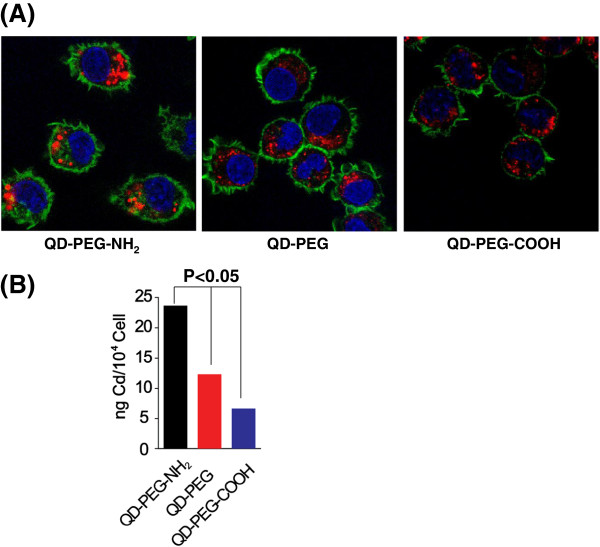
**Localization of QDs in J774A.1 cells. (A)** Cells after treatment with 47 μg/ml QDs for 24 h were co-stained with DAPI and FITC-conjugated phalloidin. Fluorescence for DAPI (blue), FITC (green), and QDs (red) was examined through confocal laser scanning microscopy. The three colors were merged together. Original magnification, ×400. **(B)** Intracellular cadmium mass in cells after exposure to QDs with different surface modifications for 24 h was analyzed by ICP-MS (*n* = 3).

To further search for the mechanism responsible for the cytotoxicity caused by QD-PEG-NH_2_ particles, we examined the intracellular localization of QDs inside the cells. We first employed the technique of confocal microscopy to survey intracellular localization of QDs in J774A.1 cells, through staining the cytoskeleton with FITC-conjugated phalloidin (green) and nucleus with DAPI (blue). After 24-h exposure, the cells were treated as previously described [12], and fluorescence for nuclei, cytoskeleton, and QDs were visualized through confocal laser scanning microscopy. As shown in Figure 4A, QDs (in red) were observed predominantly in cytoplasm with little present in plasma membrane and nucleus similar to cells upon different treatments with QD-PEG, QD-PEG-COOH, or QD-PEG-NH_2_ particles. The intracellular intensity of QD-PEG-NH_2_ particles was brighter than that in the cells treated with QD-PEG-COOH or QD-PEG particles, indicating enhanced localization of QD-PEG-NH_2_ particles in cytoplasm (Figure 4A). To confirm this finding, we determined the total Cd mass inside the cells using ICP-MS. As shown in Figure 4B, the Cd concentration was the highest in QD-PEG-NH_2_-exposed cells compared to that in the cells treated with QD-PEG or QD-PEG-COOH (> twofold,). Increased cellular uptake of QD-PEG-NH_2_ particles could be interpreted as being caused by a high affinity between QD-PEG-NH_2_ particles and cell membrane, which promoted transportation of QDs into the cells through endocytosis and diffusion [25,26]. Therefore, the inhibition of cell proliferation by QD-PEG-NH_2_ particles presumably resided in their substantial accumulation within the cells.

**Figure 5:**
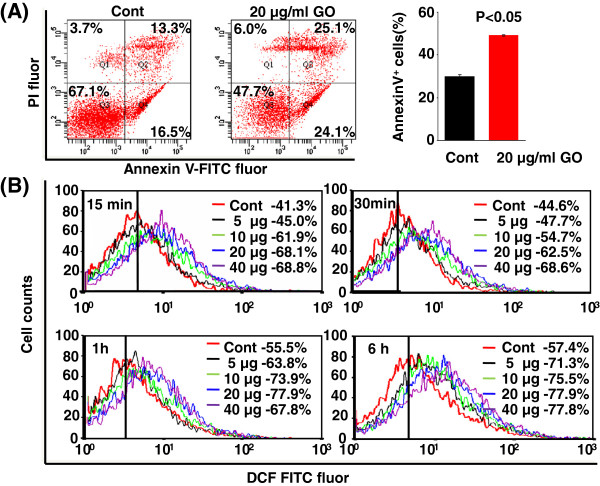
**GO-triggered cell death of erythroid cells through apoptosis. (A)** Representative FACS images describing fetal liver cell death upon GO treatment at 20 μg/ml for 24 h using Annexin V and PI staining. **(B)** FACS analysis of relative fluorescent intensity reflecting ROS content after GO exposure at various concentrations at different time points in fetal liver cells. ANOVA was used to determine the mean difference in cells treated with GO at different concentrations and along time course compared to control.

It was reported that GO exposure led to cytotoxicity to macrophages [15]. It was also documented that GO could cause hemolysis *in vitro*[13]. Thus far, the biological performance of GO on erythroid progenitor cells has not been investigated. We here assessed the impact of GO exposure on primary E14.5 fetal liver cells, which are predominantly erythroid progenitor cells with a small portion of other types of cells, such as macrophages [19,27,28]. GO provoked the substantial cell death of E14.5 fetal liver cells via apoptosis, as shown in Figure 5A, the percentages of Q4 (early apoptosis) plus Q2 (late apoptosis) were significantly increased in GO-treated cells (at 20 μg/ml, *P* < 0.05) compared to the control cells. Overall, the apoptotic cells (Annexin V^+^) increased considerably upon exposure to GO in comparison to the control cells (29.9% vs. 49.2%, Figure 5A, *P* < 0.05). It should be noted that in spite of only a small proportion of macrophages in fetal liver, they are indispensable for fetal erythropoiesis involving the establishment of erythroblastic islands [29]. We also observed a slight increase of necrosis in fetal liver cells treated with GO (Figure 5A), which was presumably due to the difference of fetal liver macrophages from erythroid cells in terms of their process of death (i.e., necrosis for macrophages upon GO treatment).

Our recent study suggested that sodium arsenite induced substantial oxidative stress (ROS synthesis), resulting in apoptosis on erythroid cells [30]. We therefore assessed the intracellular ROS level in fetal liver cells after GO treatment. As shown in Figure 5B, the DCF fluorescent intensity was greatly enhanced in fetal liver cells treated with GO at various concentrations for only 15 min (Figure 5B, *P* < 0.001). The clear shift of DCF fluorescent peak continued at 0.5, 1, and 6 h (Figure 5B, *P* < 0.001). These results together suggested that GO-induced apoptosis in erythroid cells was likely dependent on ROS-mediated oxidative stress, similar to the mechanism responsible for arsenic-stimulated apoptosis in erythroid cells [30]. Additionally, GO treatment was here determined to cause cell death in erythroid cells via apopotosis, similar to a study demonstrating that graphene stimulated ROS generation and induced cell death via apoptosis in PC12 cells, a cell line derived from a pheochromocytoma of the rat adrenal medulla [4].

**Figure 6:**
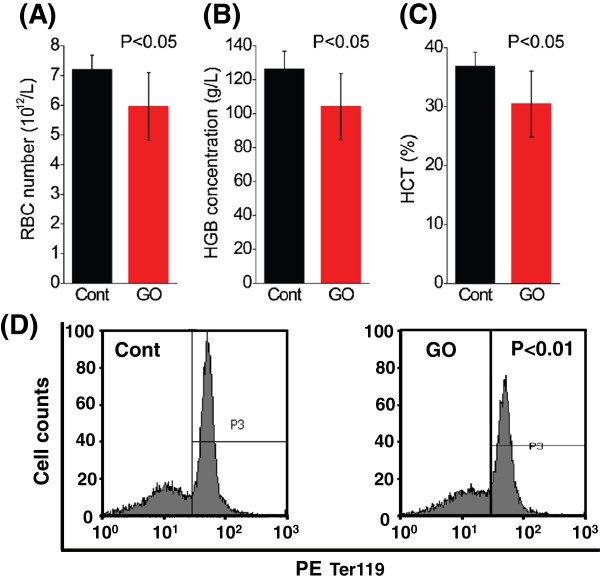
**Results of CBC indexes.** After a 3-week treatment, mice were sacrificed, and peripheral blood was collected via the heart followed by CBC analysis. **(A)** Red blood cell (RBC) counts, **(B)** hemoglobin concentration (HGB), and **(C)** hematocrit (HCT). **(D)** After mincing of spleens, the single-cell suspensions were stained with PE conjugated with Ter119^+^ to label erythroid progenitor population and were then subject to FACS analysis.

To substantiate the finding of GO-induced cell death on erythroid cells, we performed *in vivo* exposure of GO in mice. Considerable thrombus formation could be induced by intravenously injected GO, indicating that this method of exposure is not applicable for repeated administration of GO in evaluating its death-inducing effect on blood cells [18,31]. Thus, in the current study, intraperitoneal injection was selected for GO treatment in mice. No mortality in any group was found, and no signs of gross toxic symptoms (such as body weight loss and abnormal activity or diet) were observed (data not shown). The CBC analysis indicated that the RBC number in peripheral blood was reduced by 17% in GO-exposed mice compared to the control mice (Figure 6A, *P* < 0.05), accompanied by a significant decrease of hemoglobin (HGB) concentration (Figure 6B, *P* < 0.05) and hematocrit (HCT) (Figure 6C, *P* < 0.05). These results suggested that GO treatment greatly impaired RBCs, leading to a reduced number in peripheral blood, and also supported the finding of GO-mediated cell death on erythroid cells (Figure 5).

**Figure 7:**
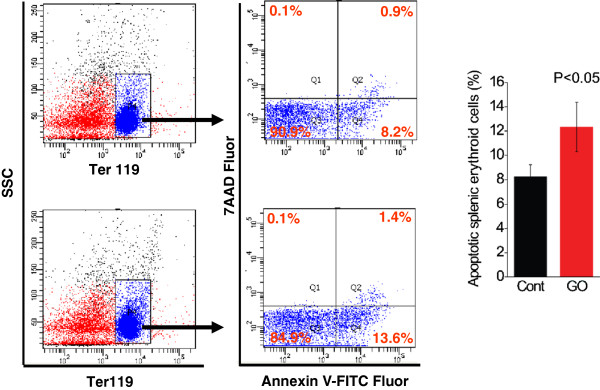
**GO-promoted cell death of splenic erythroid cells.** FACS analysis of proportion of apoptotic erythroid cells (Ter119^+^ cell population). The single-cell suspensions from spleens were simultaneously stained with PE-conjugated anti-Ter119 Ab, FITC-conjugated Annexin V, and 7AAD to sort the apoptotic Ter119^+^ in spleens. After sorting in the first left gate, Ter119 positive cells were selected and then further analyzed for cell death. The quantified data for the average percentage of apoptotic Ter119^+^ cells are shown in the bar graph (*n* = 4).

To validate the effect of GO on the survival of erythroid cells, we further investigated the cell death of erythroid cells from spleen. Since bone marrow and spleen are active sites of erythropoiesis in early course, we looked at the proportion of erythroid cells in spleen and bone marrow with FACS analysis. As shown in Figure 6D, there was a significant reduction (approximately 10%) of Ter119^+^ population (representing erythroid cells) in spleens from mice administrated with GO compared to the control (*P* < 0.05), indicating that GO exposure diminished erythroid cells in spleen. To substantiate this observation, we assessed the cell death of Ter119^+^ cells by simultaneously staining the splenic cells with PE-conjugated anti-Ter119 Ab, FITC-conjugated Annexin V, and 7AAD [30]. Similar to PI, 7AAD was used to label necrotic dead cells. Under the FACS analysis, Ter119^+^ cells were selected for the determination of cell death with Annexin V and 7AAD (Figure 7). Compared to the control mice, there was a significant increase in the percentage of apoptotic Ter119^+^ cells in spleens from the GO-exposed mice (Figure 7, *P* < 0.05). However, these changes of a decrease in the erythroid cell population and an increase of erythroid apoptosis were not observed in the bone marrow (data not shown). These findings revealed that GO exposure could result in a great reduction of splenic erythroid cells through apoptosis but not for bone marrow erythroid cells. The large difference between spleen and bone marrow is likely due to a very difficult transportation of GO into the bone marrow through circulation and a higher sensitivity to apoptosis of erythroid progenitors in spleen than those in bone marrow as well [22,32]. Together, these findings demonstrated that GO greatly impaired erythroid population through inducing cell death of erythroid cells.

## Conclusions

The blood circulation system is an important barrier against invaders, including nanomaterials under biomedical applications or environmental absorption. The blood cells are primarily responsible for governing their trafficking and systemic translocation. Since RBCs are the most abundant cell population in peripheral blood (4.1 to 5.9 × 10^6^/ml RBCs vs. 4.4 to 11.3 × 10^6^/ml white blood cells in humans), these cells presumably have a much greater probability of exposure to nanomaterials in the circulation after administration, with possible adverse effects such as hemolysis [33-35]. For clearance of nanomaterials from the circulation, the macrophages are responsible for recognizing and ingesting these particles [36]. Therefore, the nanomaterials transporting in the circulation or deposited within macrophages could cause harm to these cells as well as to the immune system. To date, studies on toxicity of QDs and GO to RBCs or macrophages have been limited and without conclusive answers, and this certainly warrants detailed investigation.

Our combined results demonstrated that QDs could be readily engulfed by macrophages and provoked intracellular ROS generation. Particularly, QDs coated with PEG-NH_2_ had a greater capability for entering the cells and revealed a robust ability to repress the proliferation of J774A.1 cells. This indicated that surface modification could be optimized to ensure the function and the safety of QDs as well. Meanwhile, to the best of our knowledge, the biological impact of graphene on erythroid progenitor cells has not been previously reported. Our study is the first to demonstrate that GO could provoke apoptosis of erythroid cells *in vitro* and *in vivo*. These data suggested that GO could likely possess the potential to disrupt the concerted balance of erythropoiesis in mammalians including humans. Thus, the adverse effects of GO on RBCs warranted further detail investigation, especially for humans under biomedical and environmental exposure.

## Competing interests

The authors declare that they have no competing interests.

## Authors’ contributions

GQ and SL conceived and designed the study. GQ, XW, ZW, and SL carried out the experiments, and GQ and SL analyzed the data. GQ and SL wrote the paper. All authors read and approved the final manuscript.

## References

[B1] Pelley JL Daar AS Saner MA State of academic knowledge on toxicity and biological fate of quantum dots Toxicol Sci 2009 8 2 276 296 10.1093/toxsci/kfp188 19684286 PMC2777075

[B2] Yong KT Law WC Hu R Ye L Liu L Swihart MT Prasad PN Nanotoxicity assessment of quantum dots: from cellular to primate studies Chem Soc Rev 2012 8 3 1236 1250 10.1039/c2cs35392j 23175134

[B3] Chang YL Yang ST Liu JH Dong E Wang YW Cao AN Liu YF Wang HF In vitro toxicity evaluation of graphene oxide on A549 cells Toxicol Lett 2011 8 3 201 210 10.1016/j.toxlet.2010.11.016 21130147

[B4] Zhang YB Ali SF Dervishi E Xu Y Li ZR Casciano D Biris AS Cytotoxicity effects of graphene and single-wall carbon nanotubes in neural phaeochromocytoma-derived PC12 Cells ACS Nano 2010 8 6 3181 3186 10.1021/nn1007176 20481456

[B5] Hardman R A toxicologic review of quantum dots: toxicity depends on physicochemical and environmental factors Environ Health Perspect 2006 8 2 165 172 10.1289/ehp.8284 16451849 PMC1367826

[B6] Tang M Xing T Zeng J Wang H Li C Yin S Yan D Deng H Liu J Wang M Chen J Ruan DY Unmodified CdSe quantum dots induce elevation of cytoplasmic calcium levels and impairment of functional properties of sodium channels in rat primary cultured hippocampal neurons Environ Health Perspect 2008 8 7 915 922 10.1289/ehp.11225 18629314 PMC2453160

[B7] Varin A Gordon S Alternative activation of macrophages: immune function and cellular biology Immunobiology 2009 8 7 630 641 10.1016/j.imbio.2008.11.009 19264378

[B8] Ganz T Nemeth E Regulation of iron acquisition and iron distribution in mammals Biochimica Et Biophysica Acta-Molecular Cell Research 2006 8 7 690 699 10.1016/j.bbamcr.2006.03.014 16790283

[B9] Nairz M Weiss G Molecular and clinical aspects of iron homeostasis: from anemia to hemochromatosis Wien Klin Wochenschr 2006 8 15–16 442 462 16957974 10.1007/s00508-006-0653-7

[B10] Hentze MW Muckenthaler MU Andrews NC Balancing acts: molecular control of mammalian iron metabolism Cell 2004 8 3 285 297 10.1016/S0092-8674(04)00343-5 15109490

[B11] Wang TT Bai J Jiang X Nienhaus GU Cellular uptake of nanoparticles by membrane penetration: a study combining confocal microscopy with FTIR spectroelectrochemistry ACS Nano 2012 8 2 1251 1259 10.1021/nn203892h 22250809

[B12] Qu GB Zhang CW Yuan L He JY Wang Z Wang LX Liu SJ Jiang GB Quantum dots impair macrophagic morphology and the ability of phagocytosis by inhibiting the Rho-associated kinase signaling Nanoscale 2012 8 7 2239 2244 10.1039/c2nr30243h 22395807

[B13] Liao KH Lin YS Macosko CW Haynes CL Cytotoxicity of graphene oxide and graphene in human erythrocytes and skin fibroblasts ACS Appl 2011 8 7 2607 2615 10.1021/am200428v 21650218

[B14] Sasidharan A Panchakarla LS Chandran P Menon D Nair S Rao CNR Koyakutty M Differential nano-bio interactions and toxicity effects of pristine versus functionalized graphene Nanoscale 2011 8 6 2461 2464 10.1039/c1nr10172b 21562671

[B15] Li Y Liu Y Fu YJ Wei TT Le Guyader L Gao G Liu RS Chang YZ Chen CY The triggering of apoptosis in macrophages by pristine graphene through the MAPK and TGF-beta signaling pathways Biomaterials 2012 8 2 402 411 10.1016/j.biomaterials.2011.09.091 22019121

[B16] Chen GY Yang HJ Lu CH Chao YC Hwang SM Chen CL Lo KW Sung LY Luo WY Tuan HY Hu YC Simultaneous induction of autophagy and toll-like receptor signaling pathways by graphene oxide Biomaterials 2012 8 27 6559 6569 10.1016/j.biomaterials.2012.05.064 22704844

[B17] Liu W Zhang SP Wang LX Qu C Zhang CW Hong L Yuan L Huang ZH Wang Z Liu SJ Jiang GB CdSe quantum dot (QD)-induced morphological and functional impairments to liver in mice PLoS One 2011 8 9 e24406 10.1371/journal.pone.0024406 21980346 PMC3182941

[B18] Qu GB Wang XY Liu Q Liu R Yin NY Ma J Chen LQ He JY Liu SJ Jiang GB The ex vivo and in vivo biological performances of graphene oxide and the impact of surfactant on graphene oxide’s biocompatibility J Environ Sci 2013 8 5 1 9 10.1016/s1001-0742(12)60252-6 24218816

[B19] Zhang J Socolovsky M Gross AW Lodish HF Role of Ras signaling in erythroid differentiation of mouse fetal liver cells: functional analysis by a flow cytometry-based novel culture system Blood 2003 8 12 3938 3946 10.1182/blood-2003-05-1479 12907435

[B20] Lok CN Ho CM Chen R He QY Yu WY Sun H Tam PK Chiu JF Che CM Silver nanoparticles: partial oxidation and antibacterial activities J Biol Inorg Chem 2007 8 4 527 534 10.1007/s00775-007-0208-z 17353996

[B21] Liu S Goldstein RH Scepansky EM Rosenblatt M Inhibition of Rho-associated kinase signaling prevents breast cancer metastasis to human bone Cancer Res 2009 8 22 8742 8751 10.1158/0008-5472.CAN-09-1541 19887617

[B22] Liu Y Pop R Sadegh C Brugnara C Haase VH Socolovsky M Suppression of Fas-FasL coexpression by erythropoietin mediates erythroblast expansion during the erythropoietic stress response in vivo Blood 2006 8 1 123 133 10.1182/blood-2005-11-4458 16527892 PMC1895827

[B23] Vanoers MHJ Reutelingsperger CPM Kuyten GAM Vondemborne AEGK Koopman G Annexin-V for flow cytometric detection of phosphatidylserine expression on B-cells undergoing apoptosis Blood 1994 8 10 A291 A291 8068938

[B24] Cho SJ Maysinger D Jain M Roder B Hackbarth S Winnik FM Long-term exposure to CdTe quantum dots causes functional impairments in live cells Langmuir 2007 8 4 1974 1980 10.1021/la060093j 17279683

[B25] Clift MJ Rothen-Rutishauser B Brown DM Duffin R Donaldson K Proudfoot L Guy K Stone V The impact of different nanoparticle surface chemistry and size on uptake and toxicity in a murine macrophage cell line Toxicol Appl Pharmacol 2008 8 3 418 427 10.1016/j.taap.2008.06.009 18708083

[B26] Zhang LW Monteiro-Riviere NA Mechanisms of quantum dot nanoparticle cellular uptake Toxicol Sci 2009 8 1 138 155 10.1093/toxsci/kfp087 19414515

[B27] Liu SJ Bhattacharya S Han A Suragani RNVS Zhao W Fry RC Chen JJ Haem-regulated eIF2alpha kinase is necessary for adaptive gene expression in erythroid precursors under the stress of iron deficiency Br J Haematol 2008 8 1 129 137 10.1111/j.1365-2141.2008.07293.x 18665838 PMC5072281

[B28] Zhang J Lee EY Liu Y Berman SD Lodish HF Lees JA pRB and E2F4 play distinct cell-intrinsic roles in fetal erythropoiesis Cell Cycle 2010 8 2 371 376 10.4161/cc.9.2.10467 20023434 PMC3931422

[B29] Kawane K Fukuyama H Kondoh G Takeda J Ohsawa Y Uchiyama Y Nagata S Requirement of DNase II for definitive erythropoiesis in the mouse fetal liver Science 2001 8 5521 1546 1549 10.1126/science.292.5521.1546 11375492

[B30] Suragani RNVS Zachariah RS Velazquez JG Liu SJ Sun CW Townes TM Chen JJ Heme-regulated eIF2 alpha kinase activated Atf4 signaling pathway in oxidative stress and erythropoiesis Blood 2012 8 22 5276 5284 10.1182/blood-2011-10-388132 22498744 PMC3369616

[B31] Singh SK Singh MK Nayak MK Kumari S Shrivastava S Gracio JJA Dash D Thrombus inducing property of atomically thin graphene oxide sheets ACS Nano 2011 8 6 4987 4996 10.1021/nn201092p 21574593

[B32] Guihard S Clay D Cocault L Saulnier N Opolon P Souyri M Pages G Pouyssegur J Porteu F Gaudry M The MAPK ERK1 is a negative regulator of the adult steady-state splenic erythropoiesis Blood 2010 8 18 3686 3694 10.1182/blood-2009-09-242487 20223923

[B33] Cheng FY Su CH Yang YS Yeh CS Tsai CY Wu CL Wu MT Shieh DB Characterization of aqueous dispersions of Fe3O4 nanoparticles and their biomedical applications Biomaterials 2005 8 7 729 738 10.1016/j.biomaterials.2004.03.016 15350777

[B34] Kainthan RK Gnanamani M Ganguli M Ghosh T Brooks DE Maiti S Kizhakkedathu JN Blood compatibility of novel water soluble hyperbranched polyglycerol-based multivalent cationic polymers and their interaction with DNA Biomaterials 2006 8 31 5377 5390 10.1016/j.biomaterials.2006.06.021 16854460

[B35] Dobrovoiskaia MA Clogston JD Neun BW Hall JB Patri AK McNeil SE Method for analysis of nanoparticle hemolytic properties in vitro Nano Lett 2008 8 8 2180 2187 10.1021/nl0805615 18605701 PMC2613576

[B36] Dobrovolskaia MA McNeil SE Immunological properties of engineered nanomaterials Nat Nanotechnol 2007 8 8 469 478 10.1038/nnano.2007.223 18654343

